# Proteasomal activity and disease outcome in phenylketonuria patients with a structural SLC7A5 variant

**DOI:** 10.1038/s41598-025-31622-w

**Published:** 2025-12-12

**Authors:** Miroslaw Bik-Multanowski, Sylwia Bobis-Wozowicz, Marcin Piejko, Anna Madetko-Talowska, Katarzyna Szewczyk, Kinga Kopaczka-Koziol, Slawomir Lasota, Urszula Jankowska, Bozena Skupien-Rabian, Dorota Korycinska-Chaaban, Amanda Krzywdzinska-Rogowska, Ortrud K. Steinlein

**Affiliations:** 1 Institute of Human Genetics, University Hospital, LMU Munich, Munich, Germany; 2 Department of Medical Genetics, Jagiellonian University Medical College, Krakow, Poland; 3 Jagiellonian University, Faculty of Biochemistry, Biophysics and Biotechnology, Department of Cell Biology, Krakow, Poland; 4 Jagiellonian University Medical College, Krakow, Poland; 5 Jagiellonian University, Faculty of Biochemistry, Biophysics and Biotechnology, Proteomics Core Facility, Krakow, Poland; 6 Jagiellonian University, Malopolska Centre of Biotechnology, Krakow, Poland; 7 Department of Inborn Errors of Metabolism and Paediatrics, Institute of Mother and Child, Warsaw, Poland

**Keywords:** PKU, Intelligence, Cellular model, FOXO pathway, Proteasomes, Cancer, Genetics, Molecular biology

## Abstract

Treatment in phenylketonuria is based on using a diet aimed at protecting the brain from uncontrolled hyperphenylalaninemia. Although treatment methods are well established, clinical outcomes vary among patients. This may be due to alterations of phenylalanine transport mediated by the amino acid transporter LAT1, which also regulates the function of the mTORC1/proteasomal pathway. We investigated the clinical and cellular effects of a structural variant in the *SLC7A5* gene, which encodes LAT1. The investigated variant was previously associated with altered phenylalanine metabolism in preliminary studies. We assessed physical and intellectual development in children with phenylketonuria. Next, we explored the cellular mechanisms underlying potential phenotypic differences between carriers and noncarriers of the rs113883650 polymorphism – a marker of the studied *SLC7A5* variant. For cellular experiments we used a model of hyperphenylalaninemia based on induced pluripotent stem cells. We assessed LAT1 abundance and performed transcriptomic and proteomic analyses. We found that carriers of rs113883650 were more prone to being overweight but exhibited significantly better intellectual development. They also showed a corresponding increased LAT1 abundance and decreased expression of proteasomal genes and the FOXO signalling pathway. We propose considering of the rs113883650 status during the follow-up of children with phenylketonuria. It may also hold relevance in other LAT1-related conditions including cancers.

**Supplementary Information:**

The online version contains supplementary material available at 10.1038/s41598-025-31622-w.

## Introduction

Phenylketonuria (PKU; MIM #261600) is a classic example of a treatable inborn error of metabolism. The major biochemical sign of PKU is massive hyperphenylalaninemia resulting from deficient activity of the enzyme phenylalanine hydroxylase (encoded by the *PAH* gene) in the liver^1^. The disease can be effectively treated with a lifelong low-phenylalanine diet. The diet should be introduced immediately after obtaining a positive result in PKU screening, which is routinely performed for newborns.

The goal in this context is to minimize the hyperphenylalaninemia and thus avoid brain damage related to hyperphenylalaninemia. This can be done by keeping the concentration of phenylalanine (Phe) in the blood within a range that is considered safe, although it may exceed physiological levels. It is generally accepted that such a therapeutic regimen promotes normal intellectual development in patients with PKU^1–3^.

Predicting the clinical course of the disease based on *PAH* genotype is not straightforward, even for patients with a complete absence of phenylalanine hydroxylase activity^4–6^. One of the reasons for this is treatment-adherence problems. Proper dietary treatment requires a high level of commitment and consistency as it involves the near-total elimination of food products containing natural protein and a parallel supplementation of special Phe-free amino-acid formula several times a day^7,8^. Treatment adherence can be assessed for a patient using their median/mean blood concentration of Phe or the percentage of control measurements of blood Phe with Phe below the maximal tolerated concentration.

It should be noted, however, that nearly all patients with PKU go through periods of high and labile blood phenylalanine. This is due to not only dietary inconsistencies, but also irregularities in lifestyle or various health issues with increased catabolism and increased endogenous production of Phe. Nevertheless, the number and length of such periods should be kept to a minimum by frequent blood monitoring for Phe and prompt adjustment of the low-Phe diet to individual metabolic fluctuations.

Short-term hyperphenylalaninemia (lasting days) can result in reversible cognitive dysfunction, including decreased executive function, reduced attention span, or worsened working memory^8–12^. Prolonged and recurrent periods of massive hyperphenylalaninemia are widely believed to result in irreversible brain damage with intellectual disability, neurological disorders, and psychiatric issues. The exact pathomechanism of brain dysfunction in PKU is not entirely clear, but neurotransmitter fluctuations (especially dopamine deficits) and alteration of protein synthesis in the brain due to amino acid imbalance are believed to play critical roles^13^. PKU complications also include extracerebral issues such as obesity and arterial hypertension^14^. Nevertheless, some patients seem to be “protected” and reveal only mild or even a lack of cognitive dysfunction despite long-term exposure to significant hyperphenylalaninemia^15–20^.

Although the exact mechanism of toxicity in hyperphenylalaninemia remains unclear, it is widely accepted that the large neutral amino acid transporter type 1 (LAT1) plays a significant role^21–23^. LAT1 is the primary transmembrane transporter for Phe, other large neutral amino acids, and their derivates (e.g., thyroid hormones, L-DOPA, melphalan, and gabapentin)^24,25^. The LAT1-mediated influx of amino acids is also a key regulatory mechanism of the mTORC1 pathway, which is crucial for controlling protein degradation via the ubiquitin proteasome system. Additionally, the cellular activity of proteasomes can be directly influenced by Phe, as high cytoplasmic concentrations of Phe inhibit proteasome translocation from nucleus to the cytoplasm^26–28^. Therefore, LAT1 saturation by Phe may lead to competitive inhibition of the transmembrane transport of other amino acids and their derivatives, as well as disruptions in the functions of both the mTORC1 pathway and the proteasome system.

A functional LAT1 transporter is composed of two proteins that are encoded by two distinct genes: *SLC7A5* encodes the LAT1 small subunit (also known as CD98 light chain), and *SLC3A2* encodes the 4F2hc subunit (CD98 heavy subunit). The LAT1 small subunit determines the specificity and effectiveness of the amino acid transfer, whereas 4F2hc is its structural part and is bound to the cell membrane^24^. Considering the capacity of LAT1 to control the transport of Phe and subsequently alter the clinical outcome of PKU, several studies have aimed to identify functional genetic variants in *SLC7A5*. As of now, only a few rare missense variants have been found in the coding regions of this gene, and they do not have a clear potential to impact LAT1 function^22,23^.

However, in our preliminary studies, we detected an association of a common haplotype of the *SLC7A5* gene (allelic frequency: 0.22) with a tendency toward being overweight among infants with PKU and with alteration of brain Phe content in adults who have uncontrolled massive hyperphenylalaninemia^29,30^. The rs113883650 variant is a diagnostic marker of the above haplotype and involves a deletion of five nucleotides (ATATG) located in intron 3 within 28 nucleotides to the fourth exon of the gene (ENSG00000103257; position 16: 87840502–87840508).

In this study, the goal was to verify the functional role of the *SLC7A5* rs113883650 variant. We used clinical data and a cellular model of hyperphenylalaninemia based on human induced pluripotent stem cells (hiPSCs). Specifically, we investigated the potential impact of rs113883650 on long-term weight status and intellectual development in children with PKU. We also examined the effects of rs113883650 on LAT1 transporter expression, the whole transcriptome profile and the cellular proteome.

## Results

### BMI

Assessment of the BMI Z-scores revealed that carriers of rs113883650 were heavier on average than noncarriers (mean BMI Z-score 0.86 vs. 0.51). This difference was statistically significant (*t*(813) = 3.85, *p =* 0.0001). Detailed analysis showed that BMI was significantly higher in carriers at the ages of one year (mean BMI Z-score 0.9 vs. 0.52; *t*(171) = 2.26, *p =* 0.02), two years (mean BMI Z-score 0.59 vs. 0.11; *t*(122) = 2.6, *p =* 0.01), and three years (mean BMI Z-score 0.96 vs. 0.42; *t*(122) = 2.14, *p =* 0.03). At the age of 4–7 years, the difference was not significant. However, it should be noted that carriers of the *SLC7A5* variant in all age groups were heavier than noncarriers, who gradually had higher rates of being overweight (Table 1).

Patients from both groups had comparable disease-causing genotypes of the *PAH* gene (Table S1).

**Table 1:** Mean BMI Z-Scores in specific age groups. BMI was significantly higher in carriers of the rs113883650 variant at ages one, two and three years. At ages four to seven years, the difference remained detectable but did not reach statistical significance.

Age group	BMI Z-Scores in noncarriers	BMI Z-Scores in carriers of the rs113883650 variant of the SLC7A5 gene	Statistical significance
1 year	0.52	0.9	t(171) = 2.26, *p* = 0.02
2 years	0.11	0.59	t(122) = 2.6, *p* = 0.01
3 years	0.42	0.96	t(122) = 2.14, *p* = 0.03
4 years	0.58	0.86	t(119) = 1.17, *p* = 0.2
5 years	0.59	0.91	t(93) = 1.29, *p* = 0.1
6 years	0.83	0.95	t(95) = 0.38, *p* = 0.7
7 years	0.71	0.81	t(79) = 0.58, *p* = 0.5

## Intellectual development of patients with PKU

The comparison between 42 noncarriers (mean age 10.4 years) and 39 carriers of the rs113883650 variant (mean age 10.7 years; including eight homozygous individuals) revealed a significantly higher intelligence quotient (IQ) among carriers (mean: − 0.28 SD vs. +0.19 SD). This difference was statistically significant (*t*(79) = 2, *p =* 0.04) and the carriers showed higher mean scores on both intelligence tests (Table 2). A direct comparison between noncarriers and the individuals homozygous for the rs113883650 variant revealed an even bigger IQ difference (mean: − 0.28 SD vs. +0.54 SD). This difference remained statistically significant (*t*(48) = 2, *p* = 0.04).

No significant differences were observed between the groups regarding historical median phenylalanine (Phe) concentrations during the first six years of life (*p =* 0.06). The median Phe levels were 250 µmol/l (range: 180–770 µmol/l) among carriers and 310 µmol/l (range: 180–730 µmol/l) among noncarriers. Similarly, the mean percentage of blood Phe test results within the target range was 47% among carriers and 41% among noncarriers, but the difference was not statistically significant (*p =* 0.3).

**Table 2:** Data on patients in whom intelligence tests were applied. Higher mean IQ values were observed in homozygous carriers of the rs113883650 variant (+ 0.54 SD) and in the overall group of carriers (+ 0.19 SD), compared to noncarriers (-0.28 SD). These differences were statistically significant (*p* = 0.04). No statistically significant age differences were found between carriers and noncarriers. * w/w = noncarriers; het = heterozygous carrier of the rs113883650 variant; homoz = patient homozygous for rs113883650; ** not known; *** according to literature data^52^.

ID	Sex	SLC7A5 genotype*	PAH genotype**	Age	Test result	Standard deviation	Test type / populational mean; SD***
NK	m	w/w	p.(Arg408Trp)/p.(Arg408Trp)	6	116	0.3	WISC-R / 112.5; 11.6
PG	f	w/w	p.(Arg408Trp)/p.(Arg408Trp)	6	107	− 0.47	WISC-R / 112.5; 11.6
SM	f	w/w	p.(Arg408Trp)/p.(Arg408Trp)	6	114	0.13	WISC-R / 112.5; 11.6
BM	f	w/w	p.(Arg408Trp)/p.(Arg408Trp)	7	126	1.16	WISC-R / 112.5; 11.6
BB	m	w/w	p.(Arg408Trp)/p.(Arg408Trp)	7	130	1.51	WISC-R / 112.5; 11.6
HK	m	w/w	p.(Arg408Trp)/p.(Arg408Trp)	7	86	− 2.28	WISC-R / 112.5; 11.6
NM	m	w/w	p.(Arg408Trp)/p.(Arg158Gln)	7	104	− 0.73	WISC-R / 112.5; 11.6
OT	m	w/w	p.(Arg408Trp)/p.(Arg408Trp)	8	124	0.99	WISC-R / 112.5; 11.6
SI	m	w/w	p.(Arg408Trp)/p.(Pro281Leu)	8	110	− 0.22	WISC-R / 112.5; 11.6
GAB	f	w/w	p.(Tyr414Cys)/N	8	99	− 1.16	WISC-R / 112.5; 11.6
FP	m	w/w	p.(Arg408Trp)/p.(Arg408Trp)	9	91	− 1.85	WISC-R / 112.5; 11.6
KK	f	w/w	p.(Arg408Trp)/p.(Arg408Trp)	9	101	− 0.99	WISC-R / 112.5; 11.6
PD	f	w/w	p.(Arg408Trp)/p.(Arg408Trp)	9	113	0.04	WISC-R / 112.5; 11.6
PW	f	w/w	p.(Arg158Gln)/p.(Pro281Leu)	9	106	− 0.56	WISC-R / 112.5; 11.6
PN	m	w/w	p.(Arg408Trp)/c.1066-11G > A	9	100	− 1.08	WISC-R / 112.5; 11.6
BW	f	w/w	p.(Arg408Trp)/c.1066-11G > A	10	115	0.22	WISC-R / 112.5; 11.6
GM	m	w/w	p.(Arg408Trp)/p.(Arg408Trp)	10	110	− 0.22	WISC-R / 112.5; 11.6
SYL	f	w/w	p.(Arg408Trp)/p.(Glu280Lys)	10	102	− 0.91	WISC-R / 112.5; 11.6
MK	f	w/w	p.(Arg408Trp)/p.(Arg408Trp)	11	133	1.77	WISC-R / 112.5; 11.6
RS	m	w/w	p.(Arg408Trp)/p.(Arg408Trp)	11	100	− 1.08	WISC-R / 112.5; 11.6
GP	m	w/w	p.(Arg408Trp)/p.(Arg158Gln)	11	86	− 2.28	WISC-R / 112.5; 11.6
ALE	m	w/w	p.(Arg158Gln)/p.(Glu390Gly)	11	115	0.22	WISC-R / 112.5; 11.6
KW	f	w/w	p.(Arg408Trp)/p.(Arg408Trp)	12	133	1.77	WISC-R / 112.5; 11.6
WP	m	w/w	p.(Arg408Trp)/c.1315 + 1G > A	12	112	− 0.04	WISC-R / 112.5; 11.6
NA	m	w/w	p.(Arg408Trp)/p.(Arg408Trp)	12	136	2.03	WISC-R / 112.5; 11.6
SA	f	w/w	p.(Arg408Trp)/p.(Arg408Trp)	13	110	− 0.22	WISC-R / 112.5; 11.6
TK	m	w/w	p.(Arg408Trp)/p.(Arg408Trp)	13	111	− 0.13	WISC-R / 112.5; 11.6
KA	f	w/w	p.(Arg408Trp)/p.(Arg408Trp)	14	124	0.99	WISC-R / 112.5; 11.6
RK	f	w/w	p.(Arg408Trp)/p.(Arg408Trp)	14	107	− 0.47	WISC-R / 112.5; 11.6
BG	m	w/w	p.(Arg408Trp)/p.(Arg408Trp)	14	91	− 1.85	WISC-R / 112.5; 11.6
CW	f	w/w	p.(Arg408Trp)/c.1315 + 1G > A	15	113	0.04	WISC-R / 112.5; 11.6
ME	m	w/w	p.(Arg408Trp)/p.(Arg408Trp)	15	107	− 0.47	WISC-R / 112.5; 11.6
WK	m	w/w	p.(Arg408Trp)/p.(Arg408Trp)	15	104	− 0.73	WISC-R / 112.5; 11.6
PAT	f	w/w	N/N	16	104	0.3	WISC-R / 112.5; 11.6
					Mean = 110	Mean = − 0.22	
WIT	m	w/w	p.(Arg408Trp)/p.(Arg408Trp)	6	100	− 0.2	IDS-2 / 102.46; 12
ZOF	f	w/w	p.(Arg408Trp)/p.(Arg408Trp)	8	95	− 0.62	IDS-2 / 102.46; 12
HUB	m	w/w	p.(Arg408Trp)/c.1066-11G > A	9	89	− 1.12	IDS-2 / 102.46; 12
MIC	m	w/w	c.1066-11G > A/N	10	77	− 2.12	IDS-2 / 102.46; 12
JUL	f	w/w	p.(Arg408Trp)/N	10	89	− 1.12	IDS-2 / 102.46; 12
IDA	f	w/w	N/N	12	119	1.38	IDS-2 / 102.46; 12
OLI	m	w/w	c.1315 + 1G > A/N	12	98	− 0.37	IDS-2 / 102.46; 12
MAT	m	w/w	p.(Arg408Trp)/p.(Arg158Gln)	15	87	− 1.29	IDS-2 / 102.46; 12
					Mean = 94.2	Mean = − 0.68	
PB	m	het	p.(Arg408Trp)/p.(Arg408Trp)	7	110	− 0.22	WISC-R / 112.5; 11.6
KD	m	het	p.(Arg408Trp)/p.(Thr323Ile)	7	94	− 1.59	WISC-R / 112.5; 11.6
RM	f	het	p.(Arg408Trp)/p.(Arg408Trp)	8	106	− 0.56	WISC-R / 112.5; 11.6
JN	f	het	p.(Arg408Trp)/p.(Arg408Trp)	9	109	− 0.3	WISC-R / 112.5; 11.6
LN	f	het	p.(Arg408Trp)/p.(Arg408Trp)	9	82	− 2.63	WISC-R / 112.5; 11.6
ŚB	m	het	p.(Arg408Trp)/p.(Arg408Trp)	10	122	0.82	WISC-R / 112.5; 11.6
AM	m	het	p.(Arg408Trp)/p.(Arg408Trp)	11	107	− 0.47	WISC-R / 112.5; 11.6
JD	m	het	p.(Arg408Trp)/p.(Arg408Trp)	11	133	1.77	WISC-R / 112.5; 11.6
MM	m	het	p.(Arg408Trp)/p.(Arg408Trp)	11	111	− 0.13	WISC-R / 112.5; 11.6
WK	f	het	p.(Arg408Trp)/p.(Arg408Trp)	11	104	− 0.73	WISC-R / 112.5; 11.6
DN	f	het	p.(Arg408Trp)/p.(Arg243Gln)	11	127	1.25	WISC-R / 112.5; 11.6
GA	f	het	p.(Arg408Trp)/p.(Leu348Val)	11	122	0.82	WISC-R / 112.5; 11.6
SM	f	het	p.(Arg408Trp)/p.(Tyr414Cys)	11	123	0.91	WISC-R / 112.5; 11.6
ŁM	f	het	p.(Arg408Trp)/p.(Arg408Trp)	12	104	− 0.73	WISC-R / 112.5; 11.6
SI	f	het	p.(Arg408Trp)/p.(Arg408Trp)	12	122	0.82	WISC-R / 112.5; 11.6
UB	m	het	p.(Arg408Trp)/p.(Arg408Trp)	12	103	− 0.82	WISC-R / 112.5; 11.6
ZD	f	het	p.(Arg408Trp)/p.(Pro281Leu)	12	115	0.22	WISC-R / 112.5; 11.6
GN	f	het	p.(Arg408Trp)/p.(Pro407Leu)	13	123	0.91	WISC-R / 112.5; 11.6
OM	m	het	IVS10-11 g-a/p.(Arg252Trp)	13	134	1.85	WISC-R / 112.5; 11.6
ŹA	f	het	p.(Arg408Trp)/p.(Arg408Trp)	13	113	0.04	WISC-R / 112.5; 11.6
ŁŁ	m	het	p.(Arg408Trp)/p.(Arg158Gln)	13	115	0.22	WISC-R / 112.5; 11.6
NP	m	het	p.(Arg408Trp)/p.(Arg408Trp)	14	136	2.03	WISC-R / 112.5; 11.6
SM	m	het	p.(Arg408Trp)/c.970-2a > c	14	121	0.73	WISC-R / 112.5; 11.6
PN	f	homoz	p.(Arg408Trp)/p.(Arg408Trp)	9	124	0.99	WISC-R / 112.5; 11.6
OJ	f	homoz	p.(Arg408Trp)/p.(Arg408Trp)	9	127	1.25	WISC-R / 112.5; 11.6
SJ	m	homoz	p.(Arg408Trp)/p.(Ile283Phe)	14	111	− 0.13	WISC-R / 112.5; 11.6
PK	m	homoz	p.(Arg408Trp)/p.(Arg408Trp)	15	119	0.56	WISC-R / 112.5; 11.6
					Mean = 115.4	Mean = 0.25	
HAN	f	het	c.1066-11G > A/p.(Arg261Gln)	6	108	0.46	IDS-2 / 102.46; 12
PAW	m	het	p.(Arg408Trp)/N	8	119	1.38	IDS-2 / 102.46; 12
ALA	m	het	p.(Arg408Trp)/N	8	88	− 1.2	IDS-2 / 102.46; 12
JUL	m	het	p.(Arg408Trp)/p.(Gly272*)	8	91	− 0.96	IDS-2 / 102.46; 12
OLI	m	het	p.(Arg408Trp)/c.1066-11G > A	9	109	0.54	IDS-2 / 102.46; 12
IGO	m	het	p.(Arg408Trp)/p.(Arg158Gln)	9	112	0.79	IDS-2 / 102.46; 12
JUL	f	het	p.(Arg408Trp)/p.(Arg408Trp)	11	91	− 0.96	IDS-2 / 102.46; 12
WOJ	m	het	p.(Arg408Trp)/p.(Arg158Gln)	15	89	− 1.12	IDS-2 / 102.46; 12
KOR	f	homoz	p.(Arg408Trp)/p.(Gly272*)	6	108	0.46	IDS-2 / 102.46; 12
SAN	f	homoz	p.(Arg408Trp)/p.(Gly272*)	9	102	− 0.04	IDS-2 / 102.46; 12
HEL	f	homoz	p.(Arg408Trp)/N	11	101	− 0.12	IDS-2 / 102.46; 12
JUL	f	homoz	p.(Arg408Trp)/p.(Arg408Trp)	16	119	1.38	IDS-2 / 102.46; 12
					Mean = 103.1	Mean = 0.05	

## Expression of LAT1 subunits

**Fig. 1:**
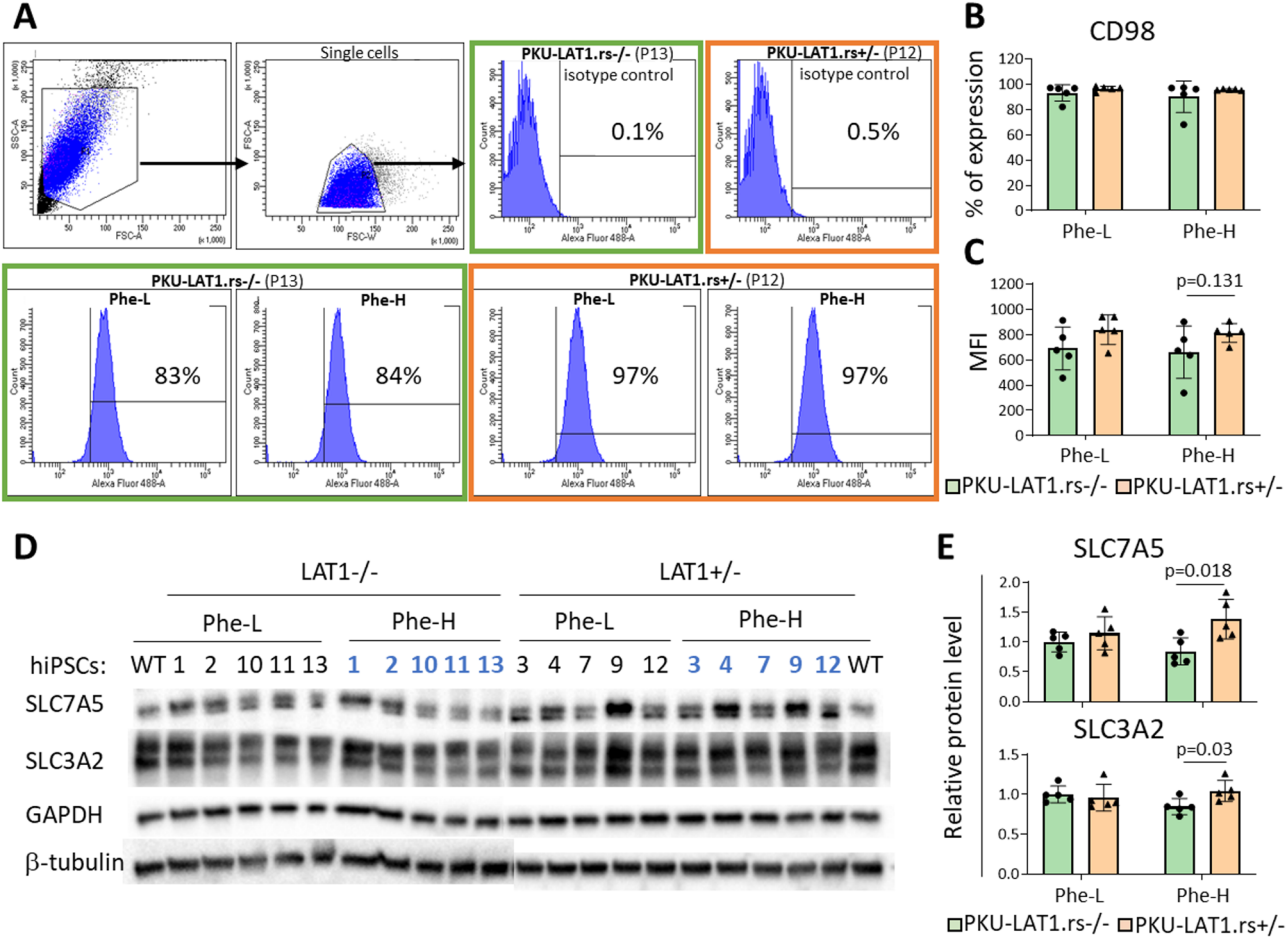
Analyses of hiPS-PKU cellular model using flow cytometry and western blot. (**A**) Representative histograms showing gating strategy and the analysis of CD98 expression by flow cytometry. (**B**) Percentage of expression of CD98. (**C**) Median fluorescence intensity (MFI) of CD98 (Phe-L: phenylalanine concentration of 215 µmol/l; Phe-H: phenylalanine concentration of 600 µmol/l) (**D**) Detection of SLC7A5 and SLC3A2 proteins in hiPS-PKU cells by western blot relative to GAPDH and β-tubulin. (**E**) Densitometric analysis of SLC7A5 and SLC3A2 proteins using Quantity One software. Statistically significant differences between noncarriers (PKU-LAT1.rs-/-) and carriers of the rs113883650 variant (PKU-LAT1.rs+/-) were detected using a *t* test for both the SLC7A5 (*p* = 0.018) and SLC3A2 (*p* = 0.03) proteins at a phenylalanine concentration of 600 µmol/l

We measured the surface expression of LAT1 protein by flow cytometry based on the presence of CD98 heavy subunit. The data showed that all hiPSC lines expressed CD98 receptor at a high percentage under all conditions tested (93 ± 6% vs. 96 ± 2.9% at 215 µmol/l Phe and 90 ± 12.5% vs. 95.6 ± 0.5% at 600 µmol/l Phe for noncarriers and carriers, respectively; Fig. 1A, B).

Notably, when we measured the median fluorescence intensity (MFI), we detected a clear difference in the receptor abundance between the rs113883650-non carrier and rs113883650-carrier cell groups at the high dose of Phe (Fig. 1C). Thus, we then analyzed the protein levels of both LAT1 subunits using western blot. The results showed significantly higher expression level of the *SLC7A5*-encoded protein (*t*(8) = 2.9, *p* = 0.018) in rs113883650-carrier hiPSCs (1.38 ± 0.3) in comparison to non-carrier cells (0.8 ± 0.2) at high Phe concentration (Figs. 1D, E, upper panel, Fig. S2). Similarly, we detected significantly upregulated expression of the *SLC3A2*-encoded protein (*t*(8) = 2.6, *p* = 0.03) in rs113883650-carrier cells treated with high Phe concentration (1.04 ± 0.13 in carriers vs. 0.85 ± 0.1 in noncarriers; Figs. 1D, E, lower panel, Fig. S2).

## Transcriptomic analysis

In the assessment of transcriptome expression in cells treated with higher Phe concentration (600 µmol/l), we removed one of the samples from the analysis due to low quality of the raw data (inconsistency of the spatial distribution of the median signal across the array). Eventually, we analyzed the expression of the transcriptome in nine samples (five rs113883650 carrier cells and four noncarrier cells). The comparison of both groups revealed 312 transcripts that were significantly upregulated and 976 transcripts that were significantly downregulated in carriers when using an uncorrected *t* test. However, the differences in the expression of individual transcripts were not statistically significant after applying the Benjamini–Hochberg correction. On contrary, the subsequent pathway-enrichment analysis revealed in the carrier-cells a significant downregulation of 18 out of 46 transcripts of the proteasome pathway. This difference was statistically highly significant (*p <* 0.00000 after applying the Benjamini–Hochberg correction), which suggests a decreased proteasomal activity in patients with PKU who have the rs113883650 variant in the condition of hyperphenylalaninemia requiring dietary adjustment (600 µmol/l).

**Fig. 2:**
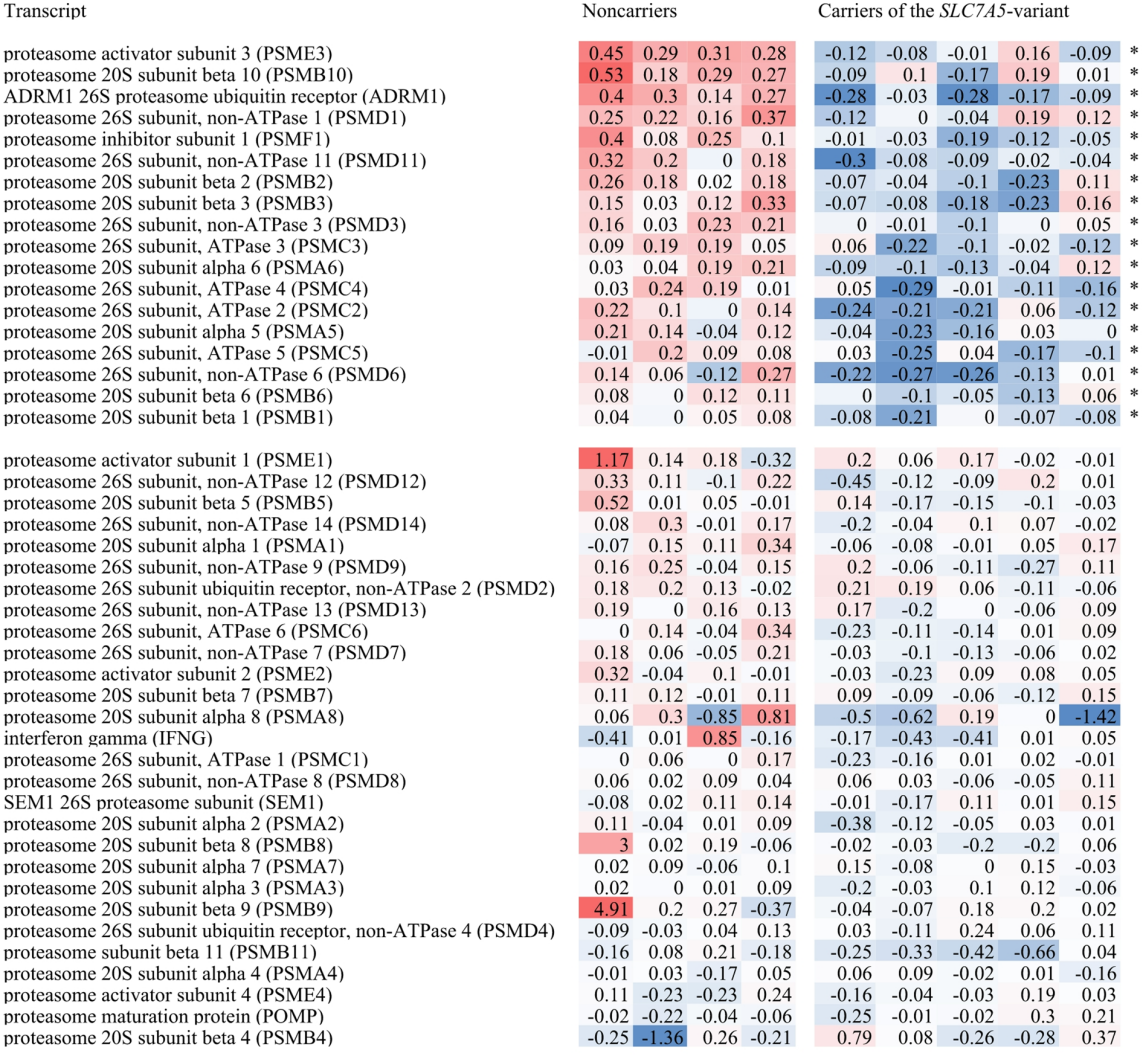
Expression of transcripts from the proteasome pathway (Kyoto Encyclopedia of Genes and Genomes) in the condition of high Phe concentration (600 µmol/l). A highly significant downregulation of the proteasome pathway was detected in the carriers of the rs113883650 variant of the SLC7A5 gene (*p* = 0.00000 in *t* test with Benjamini-Hochberg correction for multiple comparisons). *Statistically significant difference.

Figure 2 presents the expression of genes contributing to the proteasome signaling pathway in cells treated with Phe at a concentration of 600 µmol/l.

The initial comparison of whole transcriptome expression between rs113883650-carrier and rs113883650-non carrier cells treated with Phe at a concentration of 215 µmol/l (uncorrected *t* test) revealed 270 transcripts that were significantly upregulated and 691 transcripts that were significantly downregulated in carriers. However, the differences were not statistically significant after applying the Benjamini–Hochberg correction for multiple comparisons. A genomic pathway enrichment analysis also did not reveal any statistically significant differences.

Since the proteasome is regulated at the transcriptional level by transcription factors^31–33^, we performed an additional targeted analysis of the expression of *NRF1*, *NRF2*, *PAX4*, and *FOXO1*, along with the associated signaling pathways. We detected a statistically significant downregulation of 14 out of 133 transcripts in the FOXO pathway (*p* = 0.01), including *FOXO1*, in carriers of the rs113883650 variant in cells treated with high phenylalanine (Phe) concentrations.

**Fig. 3:**
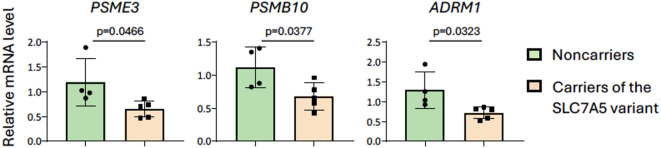
Real-time PCR validation of the transcriptomic results. The analysis confirmed the observed expression differences in relation to the top three proteasomal transcripts (*PSME3*, *PSMB10*, *ADRM1*).

To validate the microarray results, real-time PCR was performed for the first three transcripts of the proteasome pathway listed in Fig. 2 (*PSME3*, *PSMB10*, and *ADRM1*). The analysis confirmed the expression differences identified by the microarray data (Fig. 3).

## Proteomic analysis

**Fig. 4:**
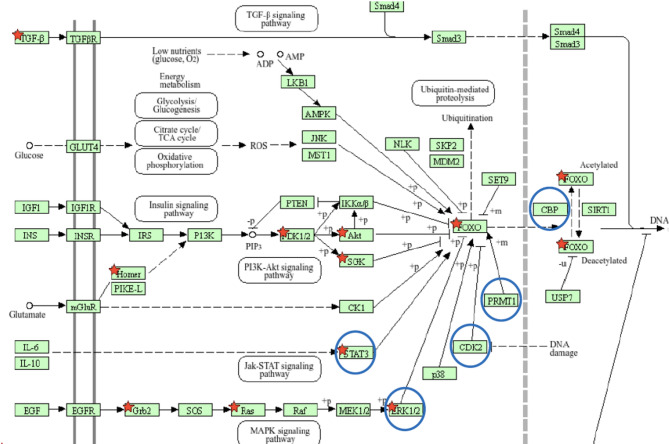
Expression of the FOXO signaling pathway in the transcriptomic and proteomic experiments.

To further support the transcriptomic findings, we conducted a targeted analysis of the expression of proteasomal components and peptides involved in the FOXO signaling pathway. This analysis confirmed a statistically significant difference in FOXO pathway expression between the two groups (*p* = 0.005, t-test) under high Phe conditions. Notably, an increased abundance of CDK2 and ERK1 (MAPK1)—both known to downregulate FOXO1—was observed in carriers of the rs113883650 variant. This finding is consistent with the results of our transcriptomic analysis (Fig. 4). In contrast, no significant differences in FOXO pathway expression were observed under low Phe conditions. Similarly, we did not observe any significant differences in proteasomal activity between the groups.

Statistically significant differences were observed only between carriers and non-carriers of the rs113883650 variant in iPSCs treated with high Phe concentrations (600 µmol/L). Differentially expressed transcripts are marked with red asterisks, while differentially expressed peptides are indicated with blue circles.

The pathway scheme was adapted from the KEGG database^34,35^.

## Discussion

The results of our study support the hypothesis of the alteration of the disease course in patients with PKU who are carriers of the common *SLC7A5* (*LAT1*) variant rs113883650. We showed that the intellectual development of patients with PKU tends to be better if they carry the variant, but they also have a higher risk of being overweight in their first years of life.

In our cellular experiments, carriers of the SLC7A5 variant exhibited hyperphenylalaninemia-related upregulation of the LAT1 transporter, accompanied by a corresponding decrease in transcriptomic activity of both the cellular proteasome complex and the FOXO signalling pathway. The FOXO pathway is a critical regulator of cellular homeostasis, controlling key processes such as cell cycle arrest, apoptosis, DNA repair, and resistance to oxidative stress^32,33^. We also observed significant differences in the expression of regulatory proteins within the FOXO pathway between carriers and non-carriers.

Normal intellectual and cognitive development is the primary treatment goal for children and adolescents with PKU. The effectiveness of PKU treatment directly depends on the extend of hyperphenylalaninemia, which is deemed to be safe for the brain if maintained within the recommended range^2,3^. On the other hand, subtle or even absent cognitive alterations in patients with long periods of hyperphenylalaninemia largely exceeding the recommended range have been reported several times for many patients^18^.

These observations provide a rationale to identify genetic factors that could explain interindividual differences in susceptibility to the toxic effects of hyperphenylalaninemia. However, research in this field can be limited by the genetic heterogeneity of the PKU population, which results in various disease severity in individual patients^5,6^. The numbers of eligible patients followed up in a typical PKU treatment center are usually small, and there are differences between therapeutic regimens, which make comparisons of patients from various populations even more problematic^36,37^.

We recruited a homogenic cohort of patients. All participants were diagnosed with a classic severe form of PKU that results in minimal tolerance of dietary phenylalanine and requires continuous dietary treatment. All patients started treatment in the first weeks of life, which minimized potential toxic effects of prolonged hyperphenylalaninemia on the brain of a newborn baby. Importantly, the intellectual development of all participants was normal.

The groups did not show significant differences in the efficiency of the dietary treatment in the first six years of life, which was measured with the median Phe concentration in blood and the percentage of blood test results exceeding the maximal recommended Phe level of 360 µmol/l. However, almost all of the patients went through short periods of higher blood Phe levels, which are typically observed in cases of incidental dietary mistakes, catabolic states (e.g., febrile infections, but also due to treatment adherence problems that typically become more frequent among older children and adolescents^7,8,10^.

In this context, it should be noted that current European recommendations for PKU accept a maximal level of hyperphenylalaninemia at 600 µmol/l for patients older than 12 years (except for pregnant women)^2^. Therefore, we did not directly compare the blood Phe levels between preschool children and adolescents in our cohort. Considering our findings, however, it is not clear whether blood Phe in the range of 360–600 µmol/l is equally safe for adolescents for both carriers and noncarriers of the *SLC7A5* variant rs113883650.

To our knowledge, the present study is the first to demonstrate a significant effect of a common genetic variant on the overall intellectual capacity in early and continuously treated children with PKU. If replicated in other populations of patients, our findings might imply a need to revise the treatment recommendations regarding the maximal acceptable levels of hyperphenylalaninemia to guarantee the optimal intellectual development in patients with PKU.

Our clinical data analysis dealt also with the risk of developing obesity, which is the most common non-neurological issue in patients with PKU^14^. It should be noted that obese children often remain obese in adulthood. While being overweight itself does not seem to be a chronic condition, obesity is a risk factor for four of the leading causes of death: coronary heart disease, type II diabetes, stroke, and cancer.

In our preliminary study, we demonstrated a tendency for young infants to be overweight if they are carriers of the investigated genetic variant^29^. However, it remained unclear whether this effect persists after infancy. Our findings confirmed the hypothesis that the tendency to become overweight or obese is present in carriers of the investigated *LAT1* variant, at least through the preschool period. The BMI was significantly higher in carriers of the rs113883650 variant during the first three years of life. Increased BMI in comparison to noncarriers remained detectable until the age of 7 years, although the difference between the groups of patients did not reach statistical significance at the age of 4–7 years.

Our cellular and molecular experiments shed some light on the possible mechanisms responsible for the observed alteration of the clinical course of PKU in carriers of the rs113883650 variant. It is generally accepted that the LAT1-mediated influx of amino acids stimulates the mTOR pathway, which increases the anabolic activity of cells^23,38,39^. Increased activity of the mTOR pathway decreases the activity of the cellular proteasomal system^26,27^. In our cellular experiment, we detected an increased amount of the SLC7A5 and SLC3A2 peptides that form the LAT1 transporter in the cytoplasm, a corresponding decrease in expression of genes encoding the proteasome system as well as decreased transcriptomic and proteomic expression of the closely related FOXO pathway in carriers of the investigated LAT1 variant. Importantly, we observed these alterations of the cellular metabolism only in cells that were treated with high concentration of Phe (600 µmol/l).

Under the assumption that the experimental Phe concentrations used reflect similar blood concentrations in patients with PKU, we can hypothesize that the functional effect of the LAT1 variant only occurs in condition of hyperphenylalaninemia that requires dietary treatment. However, the mechanisms underlying LAT1 upregulation remain unclear. Further cellular studies are necessary to elucidate this process.

We did not detect a corresponding decrease in the cytoplasmic concentration of proteins comprising the proteasome complex, which might otherwise explain the observed transcriptomic differences. Nonetheless, the mechanisms regulating proteasomal activity in the cytoplasm remain poorly understood and should be elucidated in further studies.

Better understanding of the mechanisms regulating the activity of LAT1 would be of great value for patients with mTORC1-dependent diseases^40^. For example, pharmaceuticals with the potential to decrease the LAT1-dependent influx of essential amino acids could shift the metabolic activity in cancer cells towards apoptosis and help to combat cancer. This would draw great attention to functional studies on the *SLC7A5* gene and LAT1 protein^41,42^. Studies have failed to identify any frequent gene variants in the coding sequence of the *SLC7A5* gene that alter the amino-acid sequence of the encoded protein. However, two recent genome-wide association studies demonstrated phenotypic effects of an intronic variant, rs12931876 (genomic position 16:87840574), which is located near the rs113883650 variant assessed in our study (genomic position 16:87840502–8)^43,44^. These studies observed the association of the rs12931876 variant with alteration of the transport of kynurenine and X12100 (both substances are derivatives of the amino acid tryptophane, which shares the LAT1 transporter with phenylalanine).

Moreover, another genome-wide association study observed an association of an additional closely located variant (rs11865049; genomic position 16:87840534) with the central serous chorioretinopathy^45^. These findings support the hypothesis of the regulatory potential of the third intron of the *SLC7A5* gene, where all the mentioned variants are located. Importantly, the rs113883650 variant and the rs12931876 variants were shown to be coinherited (with a nearly complete linkage disequilibrium with *r*^2^ = 0.971 and *D*’ = 1), and the rs12931876 variant is located within a binding site for the transcriptional repressor CTCF (genomic location 16:87840562–87840583)^46^. CTCF regulates the 3D structure of chromatin and is thought to be pivotal for the activity of insulators, which are sequences that block the interaction between enhancers and promoter regions^47^. Alterations of a CTCF binding sites are involved in direct regulation of gene expression and the rs12931876 variant alters the core CTCF biding motif^48^. Thus, we hypothesize that the observed phenotypic effects associated with the rs113883650 variant could be related to altered regulation of *SLC7A5* expression.

Some important limitations of our study should be mentioned. Firstly, assuming that the Phe concentrations used in our cellular model correspond with similar blood Phe concentrations in patients, it is not clear whether and to what extent the activity of the proteasomal system pathway is upregulated when blood Phe remains between the tested levels of 215 and 600 µmol/l. This topic is especially interesting and requires further studies because of the difference between treatment recommendations in most European treatment centers (Phe concentrations ranging 360–600 µmol/l are widely accepted for patients older than 12 years except for pregnant women) and American centers (the maximal accepted Phe concentration is 360 µmol/l for all patient groups)^2,3^.

Secondly, cerebral symptoms of hyperphenylalaninemia toxicity might be subtle and are not limited to the decrease of IQ^9,12,49^. Thus, a comprehensive neuropsychological assessment of larger patient cohorts would be necessary to better characterize the effect of the rs113883650 variant on intellectual development in various Phe conditions in compliant and non-compliant patients. It also seems necessary to perform studies with a cellular model of the blood–brain barrier (e.g., in iPSCs representing brain vessels, where the expression of LAT1 is remarkably high) to better characterize the pathophysiological effects of hyperphenylalaninemia in brain. Finally, since hyperphenylalaninemia inhibits the LAT1-mediated transport of thyroxin, thyroxin- related homeostasis should be studied to better understand the tendency to become overweight and obese among carriers of the rs113883650 variant. A comprehensive long-term nutritional assessment of patients from other cohorts could help to determine the potential permanent metabolic effects in carriers and noncarriers.

In conclusion, this study has demonstrated important disease-modifying effects of a common variant of the *SLC7A5* gene in patients with PKU. We also demonstrated the corresponding transcriptomic alteration in the cellular model using induced pluripotent stem cells. Bearing in mind the potential long-term clinical differences between carriers and noncarriers of this variant, we encourage genotyping of children with PKU in regard to the rs113883650 variant, as well as close clinical follow-up for this group of patients.

## Methods

### General approach

The study participants were recruited from among all interested patients with PKU who were diagnosed in 2000–2021 at two Polish PKU-treatment centers (University Children’s Hospital in Krakow and Institute for Mother and Child in Warsaw). We excluded patients with low birth weight (< 2500 g) and those with any additional chronic disease that could affect intellectual or physical development (e.g., epilepsy, hypothyroidism, or chronic diarrhea). Ultimately, a group of 234 patients were recruited. All of them started treatment in the first or second week of life and were subsequently treated according to current recommendations^2^. Informed consent was obtained from all subjects and/or their legal guardian(s).

Genotyping for the rs113883650 variant of the *SLC7A5* gene was performed using direct sequencing as described previously^23^. To answer the research questions, the carriers of the variant were compared with noncarriers in regard to clinical data. To investigate the molecular background underlying the observed phenotypic differences, a cellular model of hyperphenylalaninemia based on hiPSCs was developed.

Cellular studies were done using blood samples obtained from 10 consecutive patients with PKU (5 noncarriers and 5 carriers of the rs113883650 variant). The standard concentration of Phe in the cellular medium that we used for growing hiPSCs (215 µmol/l of Phe) reflects well-controlled hyperphenylalaninemia, and the Phe concentration of 600 µmol/l corresponds with PKU that requires adjustment of the dietary treatment. Therefore, the cells were treated with these two Phe concentrations. The Jagiellonian University Bioethics Committee approved the study procedures (Decision 1072.6120.9.2019). The study was performed in accordance with the declaration of Helsinki.

### Anthropological measurements

To assess the long-term risk of overweight/obesity, serial body mass index (BMI) measurements were recorded from the 234 study participants at the age of 1–7 years. A total of 129 noncarriers and 105 carriers of the rs113883650 variant of the *SLC7A5* gene were identified, and of 815 serial BMI measurements were retrospectively analyzed. All patients were followed at the PKU center in Krakow. The measurements of body weight and body length were conducted by hospital staff on a yearly basis. We focused only on prepubertal patients to minimize the potential bias related to intentional body-weight modification that occurs with adolescents (e.g., trying to lose weight or to put on muscle mass).

### Intellectual development

Intelligence tests were conducted with a group of 81 patients with PKU aged 6–16 years (39 girls and 42 boys) in the regional PKU centers in Krakow and in Warsaw, and the results were analyzed retrospectively. The intelligence tests were conducted in 2007–2023 by clinical psychologists who were members of the PKU treatment teams. Cognitive assessment was conducted patients using the well-established Wechsler Intelligence Scale for Children – Revised (WISC-R) for 61 patients and the Intelligence and Development Scales 2 (IDS-2) for 20 patients. The WISC-R is a standardized measure of general intellectual ability consisting of verbal and performance subtests that assess different cognitive domains, including verbal comprehension, working memory, perceptual reasoning, and processing speed^50^. The IDS-2 provides a comprehensive evaluation of cognitive abilities and developmental competencies, including fluid and crystallized intelligence, executive functioning, and visual and auditory processing^51^.

To enable comparison of the results of both tests, we assessed the individual scores expressed with standard deviations (SD) and applied the norms available in the literature^52^. To avoid a potential bias related to treatment adherence, patients from both groups were compared in regard to their historical blood Phe concentrations. Since the study participants differed in age (6–16 years), we assessed only the Phe concentrations that were recorded in their first six years of life.

### Cellular reprogramming

Blood samples were collected during routine testing. Peripheral blood mononuclear cells (PBMCs) were then isolated using a Percoll gradient centrifugation method. Cell counts and viability were assessed, and approximately 4 × 10^6^ cells were cryopreserved in 2–6 vials. Subsequently, PBMCs from PKU patients were reprogrammed into human induced pluripotent stem cells (hiPSCs) using the Cytotune-iPS 2.0 Sendai Reprogramming Kit (Invitrogen/Thermo Fisher Scientific), following the manufacturer’s protocol.

Briefly, 1 × 10^5^ PBMCs were transduced with Sendai viral vectors at a multiplicity of infection (MOI) of 5 using a vector encoding KLF4, OCT4, and SOX2 (KOS), and another vector encoding cMYC. An additional MOI of 3 was used for a vector encoding KLF4. Cells were cultured in Essential 8 Medium (Gibco) on tissue culture plates coated with Geltrex LDEV-Free, hESC-Qualified, Reduced Growth Factor Basement Membrane Matrix (Gibco/Thermo Fisher Scientific).

Between three and six colonies were isolated per patient and expanded. Molecular characterization was performed on three colonies per patient, focusing on pluripotency markers. Selected clones were passaged every four days using 0.5 mM EDTA (Gibco) at a split ratio of 1:6–8. Post-passage, cells were replated on new Geltrex-coated plates in Essential 8 Medium supplemented with 10 µM Rho-associated protein kinase (ROCK) inhibitor (Y27632; Merck) for the first 24 h. Cells were maintained in a standard humidified incubator at 37 °C with 5% CO₂ and daily media changes.

A reference hiPSC line derived from a healthy donor (Gibco, A18945) was cultured under identical conditions.

For cellular experiments, hiPSC-PKU lines were cultured under two distinct phenylalanine (Phe) concentrations: 0.215 µM and 600 µM. These correspond to recommended therapeutic and elevated Phe levels, respectively. Approximately 5 × 10^4^ cells were seeded per well in a 24-well plate two days prior to Phe supplementation. After 48 h of Phe treatment, cells were harvested for further analyses.

To assess pluripotency marker expression by immunofluorescence, hiPSCs were seeded on µ-Plate 24Well Black ID 14 mm plates (Ibidi) coated with Geltrex (Gibco/Life Technologies), three days prior to staining. Cells were cultured in Essential 8 Medium to allow well-formed but not oversized colonies. Immunostaining for SSEA4, Tra-1-60, OCT4, and SOX2 was performed using the Pluripotent Stem Cell 4-Marker Immunocytochemistry Kit (Invitrogen) following the manufacturer’s protocol.

The following secondary antibodies were used at a 1:200 dilution (provided in the kit): Alexa Fluor™ 488 goat anti-mouse IgG3, Alexa Fluor™ 555 goat anti-mouse IgM, Alexa Fluor™ 555 donkey anti-rabbit, and Alexa Fluor™ 488 donkey anti-rat. Nuclei were counterstained with NucBlue stain. Imaging was conducted using a Leica DMI6000B inverted microscope with a N PLAN 10×/0.25 dry objective. Fluorescent signals were detected with specific filter cubes, and transmitted light images were acquired using Integrated Modulation Contrast (IMC). Images were captured using a Leica DFC360FX camera and LAS X 3.4.2 software. Contrast settings were manually adjusted to fully utilize the image’s dynamic range. Fluorescence channels were shown both separately and as overlays with the IMC image.

Three hiPSC clones per patient were analyzed for the expression of pluripotency genes (OCT4, NANOG, and SOX2) via real-time quantitative PCR (RT-qPCR). One clone per patient was selected based on the optimal pluripotency profile (Fig. S1A, B). These clones were expanded and further characterized. Expression of OCT4 and SOX2 was confirmed by fluorescence imaging, along with surface markers SSEA4 and Tra-1-60 (Fig. S1C). Additionally, transcript levels of OCT4 and NANOG were compared between rs113883650 carriers and noncarriers using RT-qPCR, revealing no significant differences (Fig. S1D).

To verify genomic integrity, both hiPSCs and their parental PBMCs were analyzed using Agilent 8 × 60 K two-color oligonucleotide microarrays with CytoGenomics v5.0 software (Agilent, Santa Clara, US), as recommended. Aberrations smaller than 0.5 Mb were excluded for deletions and duplications (Figures S1E, F).

Following 48 h of Phe treatment, cells were collected for downstream protein and gene expression analyses.

### RT-qPCR

Total RNA was extracted using a Universal RNA Purification Kit (Eurx), per the manufacturer’s instructions. To remove genomic DNA contamination, samples were treated with Turbo DNAse (Ambion). Two micrograms of RNA were reverse transcribed to cDNA using the NG dART RT-PCR Kit (Eurx).

Gene expression for OCT4, SOX2, and NANOG was quantified using qPCR with PowerTrack SYBR Green Master Mix (Applied Biosystems/Thermo Fisher Scientific) on a 7500 Fast Real-Time PCR System (Applied Biosystems). Relative expression was calculated using the ΔΔCt method with 18 S RNA and β-actin as housekeeping genes. Primers used were as follows (5′–3′):

- OCT4-F: CCTTCGCAAGCCCTCATTTC; OCT4-R: TAGCCAGGTCCGAGGATCAA
- NANOG-F: ACCTCAGCTACAAACAGGTGAAG; NANOG-R: TTCTGCGTCACACCATTGCT
- SOX2-F: GGGAAAGTAGTTTGCTGCCTC; SOX2-R: CAGGCGAAGAATAATTTGGGGG
- PSME3-F: GCCGAGATTTCTCAGGTCCC; PSME3-R: CAACCTTGAGCTTCACTTCCT.
- PSMB10-F: TCCAAGACGGGGTCATTCTG; PSMB10-R: GCAGTAGATTTTGGGGGCGA
- ADRM1-F: CGAGGCAGGATGACGACC; hADRM1-R: GCCCGAAACTCCACCAAGTA.
- 18 S RNA-F: GTAACCCGTTGAACCCCATT; 18 S RNA-R: CCATCCAATCGGTAGTAGCG
- β-actin-F: AGCCTCGCCTTTGCCGA; β-actin-R: CTGGTGCCTGGGGCG.

### Flow cytometry

The surface expression of CD98 receptor was detected with FITC-labeled anti-CD98hc Monoclonal Antibody (5E5; eBioscience) and controlled with FITC-labeled Mouse IgG1 kappa Isotype Control (eBioscience). Cells were harvested with 0.5 mM ethylenediaminetetraacetic acid (EDTA), and the cell suspension was centrifuged at 200 *g* for 5 min. Next, 10^5^ cells were incubated with antibody solution in 100 µl of phosphate-buffered saline (PBS) containing 2% fetal bovine serum (FBS) for 30 min at 4 °C in the dark. After washing in PBS, the cells were collected on an LSR II flow cytometer (BD Biosciences), and data were analyzed with FACS Diva software (BD Bioscience).

### Western blot assessment

After treatment with Phe, hiPSCs were lysed in cold radio-immunoprecipitation assay (RIPA) buffer (Invitrogen/Thermo Fisher Scientific) containing protease inhibitors (Halt Protease Inhibitor Cocktail; Thermo Scientific). The protein concentration was measured with a BCA Protein Assay kit (Pierce/ThermoFisher Scientific). The lysates (30 µg of each sample) were loaded on Bis-Tris 4–15% polyacrylamide gels (SDS-PAGE; Bio-Rad) and transferred to polyvinylidene fluoride (PVDF) membranes using the Trans-Blot Turbo Transfer System (Bio-Rad) with the following parameters: 25 V, 1.3 A, 7 min.

The membranes were then blocked in the SuperBlock (TBS) Blocking Buffer (Thermo Scientific) for one hour at room temperature with gentle agitation. The membranes were stained with SLC7A5 Polyclonal Antibody (PA550485) and CD98 Polyclonal Antibody (PA582611) (both from Invitrogen) at 4 °C overnight. Next, the membranes were washed three times with Tris-buffered saline with Tween^®^ 20 (TBST) and labeled with anti-mouse HRP- conjugated secondary antibodies at a dilution of 1:2000 (Cell Signaling Technology). An equal protein loading was evaluated using mouse monoclonal IgG β-tubulin and GAPDH antibodies (both from Invitrogen, 1:2000 dilution). Finally, proteins were detected using SuperSignal West Pico PLUS Chemiluminescence Substrate (Thermo Scientific) and imaged by the Gel Doc XR + Gel Documentation System (BioRad). The signal intensity of the proteins was measured by QuantityOne software (BioRad).

#### Whole-transcriptome expression

Agilent SurePrint Human Gene Expression 60Kv3 microarrays and single-color technique were used according to the manufacturer’s protocol for whole-transcriptome expression analysis. The microarrays provide robust and comprehensive coverage of genes and transcripts sourced from the RefSeq, Ensembl, UniGene, GenBank, and LNCipedia databases to provide full coverage of the human transcriptome. GeneSpring software (Agilent) and DAVID bioinformatic resources were used for data analysis^53,54^. Statistically significant differences in the expression of single transcripts were identified with a *t* test. Pathway-enrichment analysis and gene-ontology analysis were performed using Benjamini–Hochberg correction for multiple comparisons. The primary microarray data were added to the GEO public repository and are accessible at https://www.ncbi.nlm.nih.gov/geo/query/acc.cgi?acc=GSE294755.

### Proteome expression

To quantitatively assess the differential response of carrier and noncarrier hiPSC to treatment with various concentrations of Phe, we employed the stable isotope labeling by amino acids in cell culture (SILAC) technique. For the higher Phe concentration (600 µmol/l), cells were maintained in a “heavy” medium comprising SILAC DMEM/F12 (Thermo-Fisher, Ref. A2494301) supplemented with isotopically labeled amino acids, including ^13^C_6_^15^N_2_ L-Lys (Thermo-Fisher, ref. 88209), ^13^C_6_^15^N_4_ L-Arg (Thermo-Fisher, ref. 89990). Parallel cultures were maintained under physiological Phe conditions using a “light” medium formulated with SILAC DMEM/F12 supplemented with unlabeled L-Lysine (Thermo-Fisher, Ref. 89987) and L-Arginine (Thermo-Fisher, Ref. 89989). Both cell culture media were supplemented with the Essential8 supplement (Gibco/Thermo Fisher Scientific). Cells were passaged for 5–6 cycles to ensure that heavy isotope incorporation exceeded 95%. Following this incorporation period, cells in the heavy medium were directly exposed to a higher Phe concentration (0.6 mM), while cells in the light medium were maintained at the lower Phe concentration (0.215 mM). After 48 h of exposure, all cells were lysed in lysis buffer composed of 1 M Tris with 1% SDS, and lysates were stored at -80 °C for subsequent analysis by liquid chromatography-mass spectrometry (LC-MS).

Data on the expression of the proteome were added to the repository Open Data LMU and are accessible at 10.5282/ubm/data.698.

## Supplementary Information

Below is the link to the electronic supplementary material.

Supplementary Material 1

## Data Availability

Any additional information required to reanalyze the data reported in this paper as well as all unprocessed data and supplemental information in the manuscript are available from the corresponding author (Miroslaw Bik-Multanowski) upon request. Microarray data have been deposited at GEO (GEO accession number: GSE294755), and are publicly available. Proteomic data have been deposited at the repository Open Data LMU and are also publicly available at https://doi.org/10.5282/ubm/data.698. Any additional information required to reanalyze the data reported in this paper will be available from the corresponding author upon request.
