# Supplementary material for: Proteasomal activity and disease outcome in phenylketonuria patients with a structural SLC7A5 variant
Source: Sci Rep. 2025 Dec 12;15:43813. doi: 10.1038/s41598-025-31622-w (PMC12706028; doi:10.1038/s41598-025-31622-w)
Supplement: Supplementary file 1 — Supplementary Material 1 [file 41598_2025_31622_MOESM1_ESM.docx]

**Supplementary Figures S1 and S2; Supplementary Table S1**


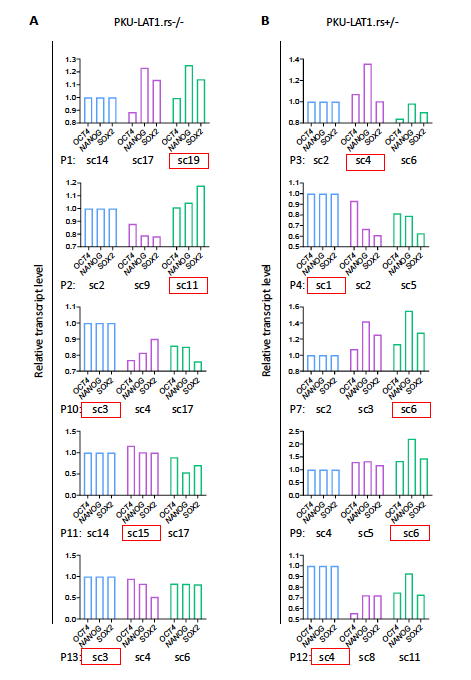


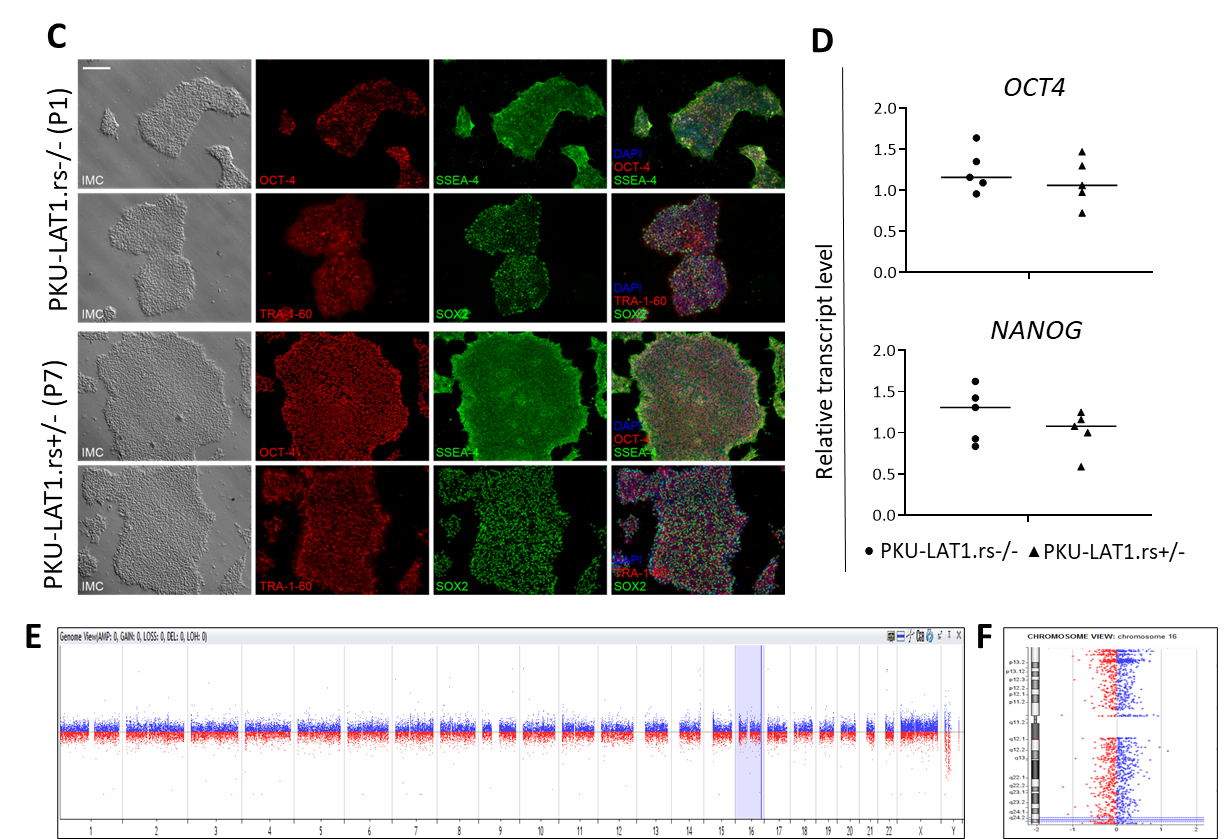


**Figure S1. Validation procedure of the cellular model. A., B.** Analysis of the expression
of pluripotency-associated transcription factors (*OCT4, NANOG, SOX2*) in single cell-derived clones (sc) of hiPS-PKU cells, noncarriers (A) and carriers (B) of the rs113883650 variant, using RT-qPCR method. **C.** Immunofluorescence analysis of the expression of pluripotency markers (OCT4, SSEA-4, Tra-1-60 and SOX2) in hiPS-PKU cells. Representative images
of noncarriers (P1) and carriers (P7) are shown. Scale bar is 150 µm. **D.** Comparison
of transcript levels of *OCT4* and *NANOG* genes in selected clones of hiPS-PKU cells, noncarriers and carriers of the rs113883650 variant. **E.** Genomic profile of copy number variants (CNVs) in hiPS-PKU and parental cells (peripheral blood mononuclear cells) detected by two-color oligonucleotide microarray Agilent 8x60K. A representative image of the whole karyotype is shown, with a blue line highlighting chromosome 16 containing the SLC7A5 gene locus. **F.** Magnification of the image of chromosome 16.

**Figure S2. Original images of the Western blot membranes shown in Figure 1D.** Two membranes containing all samples were imaged simultaneously. The dashed line indicates the cropping of the images as shown in Figure 1D. An asterisk indicates hiPSC WT treated with a phenylalanine concentration of 600 µmol/L, which was not shown in Figure 1D and was not used for densitometric calculations.

**Table S1. Genotypes of the phenylalanine hydroxylase gene (*PAH*) in patients, in whom BMI was assessed.**

| PAH genotypes | Noncarriers | Carriers of the rs113883650 variant  of the SLC7A5 gene |
| --- | --- | --- |
| p.(Arg408Trp)/p.(Arg408Trp) | 67 cases | 47 cases |
| p.(Arg408Trp)/c.1066-11g>a | 8 cases | 4 cases |
| p.(Arg408Trp)/p.(Tyr414Cys) | 7 cases | 5 cases |
| p.(Arg408Trp)/p.(Arg158Gln) | 2 cases | 3 cases |
| Other genotypes | 45 cases | 46 cases |
